# Tackling Gender and Racial Bias in Academic Emergency Medicine: The Perceived Role of Implicit Bias in Faculty Development

**DOI:** 10.7759/cureus.11325

**Published:** 2020-11-04

**Authors:** Emily C Cleveland Manchanda, Wendy L Macias-Konstantopoulos

**Affiliations:** 1 Emergency Medicine, Harvard Affiliated Emergency Medicine Residency, Boston, USA; 2 Department of Emergency Medicine, Massachusetts General Hospital, Boston, USA; 3 Department of Emergency Medicine, Harvard Medical School, Boston, USA

**Keywords:** implicit bias, faculty development, unconscious bias, diversity, inclusion, equity, gender bias, racial bias

## Abstract

Background

Gender and racial disparities in academic medicine have recently garnered much attention. Implicit Association Tests (IATs) offer a validated means of evaluating unconscious associations and preferences. This study examines the perceived role of implicit bias in faculty development in academic emergency medicine (EM).

Methods

EM faculty at a large urban academic medical center were invited to independently participate in a self-reflection assessment in preparation for a faculty retreat session discussing diversity, equity, and inclusion. Participants completed two IATs designed to examine gender associations (gender IAT) and race preferences (race IAT) followed by a short anonymous survey where IAT scores were recorded. The survey also captured demographic information and perceptions about the impact of gender and racial biases in faculty development.

Results

Forty faculty members (66%) completed the survey; 70% were male and 80% white. The majority (59%) reported gender IAT results indicating automatic male-sciences and female-liberal arts associations. Nearly half (45%) reported race IAT results indicating an automatic preference for white people. More than 70% of males reported that faculty recruitment, development, and promotion decisions were ‘never’ or ‘seldom’ affected by gender bias, while more than 80% reported racial bias ‘never’ or ‘seldom’ affects these decisions. Female faculty more frequently perceived adverse effects of unconscious gender and race biases.

Conclusion

Our group of academic physicians reported IAT results showing different levels of implicit bias compared to the general population. Female faculty may be both more aware of and more susceptible to the adverse effects of unconscious biases. Further study is needed to determine both the extent to which unconscious biases affect the academic workplace, as well as ways in which such unintentional forms of discrimination can be eliminated. Unconscious biases are not unique to EM. Intentional efforts to increase self-awareness of these 'blind spots' may help mitigate their impact and foster a more diverse and inclusive healthcare environment.

## Introduction

Background

Implicit bias is the constellation of unconscious attitudes and stereotypes that influence our thoughts, decisions, and behavior without our awareness [1]. In recent years, attention has been drawn to the adverse effects of implicit bias on both patient care outcomes [2] and patient perceptions of their doctors [3]. These unconscious thought patterns are most at play in high stress, time-pressured situations, and when operating with incomplete information [4]. As a result, emergency physicians (EPs) may be particularly susceptible to the unintended consequences of implicit biases. Special attention to this dynamic is essential in emergency departments (EDs), especially considering the diverse patient populations served and the persistent lack of diversity among ED providers in academic emergency medicine (EM) [5,6].

More recent discussions of implicit bias have expanded to include considerations of the impact of unconscious biases on the professional development, recruitment, and retention of women physicians and those from racial and ethnic groups that are underrepresented in medicine (URiM) [7,8]. The URiM designation includes physicians from any racial or ethnic background that makes up a disproportionately small segment of the physician workforce relative to their percentage in the general population [8]. Women and URiM physicians experience a myriad of challenges in the workplace related to their gender [9-11] and race/ethnicity [12,13], which are compounded in intersectional ways. Some of these challenges may stem from the unconscious biases of patients and colleagues. Consequently, implicit biases have implications beyond direct patient care, shaping work environments, and the diversity of the specialty itself.

The differential workplace experience and academic achievement of women and URiM physicians are multifactorial and are not due to implicit bias alone; structural factors including pay gaps and differential promotion despite equal academic productivity also contribute to persistent disparities [5,9,14]. Cultural factors and stereotyped expectations for interactions may also play a role, for example in negotiation, where women may be viewed as engaging in overly demanding behavior and are thus penalized for initiating conversations that might otherwise lead to more equitable pay [5,15].

Creating an inclusive and equitable work environment is complex. Faculty diversity, equity, and inclusion (DE&I) was identified as a key departmental priority in the Department of Emergency Medicine at the study institution and was one of the three topics discussed at the 2018 biennial EM faculty retreat. The overarching goals of the DE&I retreat session were to 1) encourage self-reflection and discussion regarding the role of unconscious biases in the professional development of women and URiM faculty, 2) examine departmental barriers to DE&I, and 3) identify concrete actionable strategies for improving DE&I longitudinally. A better understanding of the extent to which unconscious gender and racial biases are perceived to play a role in faculty recruitment, development, and promotion decisions could inform future DE&I initiatives and strategies. 

To promote self-reflection and stimulate discussion, we conducted a pre-retreat assessment that required faculty to complete two online Implicit Association Tests (IATs) measuring unconscious gender and racial biases, provide limited demographic information, and respond to a series of questions related to their perceptions of the role of implicit bias in professional workforce development. IATs are validated tools used to measure unconscious associations and preferences by bypassing conscious processing [16]. Despite controversy regarding the extent to which unconscious associations in this artificial test setting predict behavior, a great deal of research suggests that the IAT is a good predictor of behavior in certain contexts including medical, employment, and education outcomes [17-19].

Goals of this investigation

The primary goal of this study was to examine the perceived role of implicit bias on decisions related to professional workforce development in an academic EM department. Additionally, we aimed to assess the utility and feasibility of using IATs as an acceptable means of facilitating faculty self-reflection of unconscious biases and stimulating sensitive DE&I discussions in academic medicine.

## Materials and methods

Study design, setting, and participant recruitment

Two weeks prior to the faculty retreat in 2018, all EM faculty members at a large urban academic center received an email invitation to participate in a pre-retreat assessment. They were informed that participation was voluntary and that the assessment consisted of completing two IATs followed by a 10-minute anonymous survey. Faculty members were provided explicit instructions on how to access these free tests online, and a private, secure link to complete the survey. Faculty were not offered any specific incentive for completing the pre-retreat assessment. A reminder email was sent three days prior to the retreat encouraging faculty to complete the IATs and survey. All responses were submitted anonymously. The submission of the survey was taken as consent to participation. This study was reviewed by and exempted from further review as a quality assurance initiative by the local institutional review board (Protocol #2019P000506).

Methods of measurement

The web-based IATs are available through Project Implicit®, a non-profit organization, and international research collaboration founded in 1998. IATs chosen for this pre-retreat assessment were designed to examine; 1) association between male/female gender and the sciences vs liberal arts (gender IAT) and 2) preference for black vs white people (race IAT). Each online test presents the user with a timed dual categorization task, where they are instructed to rapidly categorize a photo or a word by pressing a left-hand or right-hand key. As the user sorts seemingly unrelated concepts, e.g. “men, father, husband” or “woman, mother, wife,” alongside words related to the sciences or liberal arts ('math, engineering' or 'music, arts'), into categories appearing in the left and right sides of the screen, the program calculates the speed with which associations are made. Response times are compared for each set of pairings. The magnitude of the difference correlates with the degree of one’s implicit bias.

The survey aimed to capture demographic information, self-reported IAT results, and perceptions regarding the extent to which implicit bias influences their work environment. A series of questions were posed related to the perceived frequency (never, seldom, occasionally, frequently, always) with which implicit bias influences decisions related to professional workforce development.

Outcome measures

Outcomes measured included the perceived frequency with which unconscious gender and racial biases affect professional workforce development, specifically in faculty recruitment, faculty proposals for academic promotion, committee participation, research collaborations, faculty nominations for leadership positions, and perceptions of faculty members’ potential for success. Secondarily, this study sought to describe the prevalence and degree of unconscious racial and gender bias, as measured by IATs, among EM faculty in our department. In addition, this study sought to demonstrate the feasibility of using IATs as a means of stimulating self-reflection about unconscious biases and sensitive DE&I discussions.

Data analysis

Descriptive statistics were used to characterize results on completed IATs and the survey. Comparisons between demographic groups based on gender and race/ethnicity were made using Pearson χ2 or Fisher’s exact test for categorical variables. Two-sample Wilcoxon rank-sum (Mann Whitney) tests were used to compare responses between binary variables and ordinal responses (e.g., the frequency with which unconscious race and gender bias affect decisions related to faculty, 5-point Likert from “Never” to “Always”). Two-sample Wilcoxon rank-sum (Mann Whitney) tests were also used to compare the distribution of our group’s self-reported IAT results with the general population’s IAT results, which are publicly available after completing an online IAT. Statistical significance was defined as P<0.05, and all tests were two-tailed. Data analysis was completed in STATA (Version 14.2, StataCorp LLC, College Station, TX).

## Results

Forty EM faculty members responded to the survey (66% response rate, see Table 1), though one individual did not report a result for the gender IAT. Eighty percent of respondents self-reported as white, and 70% of respondents were male. These proportions roughly reflect the overall makeup of our faculty group. Most respondents (80%) reported they had not previously taken an IAT; of those who had, half had taken the race IAT while the others did not recall which IAT they had taken. Almost half (47.5%) of participants reported that the results of their IATs increased their interest in overcoming unconscious biases, while 35% reported neutral feelings. The remaining 17.5% of respondents did not feel their IAT results increased their interest in overcoming unconscious biases.

**Table 1:** Demographics of survey respondents +Respondents could select multiple races/ethnicities; IAT - Implicit Association Test

	N		%		
Gender					
Male	28		70.0%		
Female	12		30.0%		
Race/Ethnicity^+^					
White or Caucasian	32		80.0%		
Asian	7		17.5%		
Pacific Islander	1		2.5%		
Hispanic	1		2.5%		
Age					
30-39	9		22.5%		
40-49	19		47.5%		
50-59	8		20.0%		
60-69	4		10.0%		
Taken an IAT previously?					
No	32		80.0%		
Yes	8		20.0%		

Gender IAT results

A total of 39 faculty members reported results for the gender IAT. The majority of respondents (59%) demonstrated some degree of automatic association between male gender and the sciences, and females with liberal arts (see Table 2). When examined by gender of the respondent, 66.7% (n=18) of male faculty but only 40% (n=5) of female faculty respondents reported an automatic association between the sciences and male gender. One-third of female faculty (n=4, 33%) and 10.7% of male faculty (n=3) respondents reported the opposite association, i.e., females with sciences, and males with liberal arts. Most respondents (57.7%) indicated they agreed with the results of their gender IAT, with roughly equal distribution across both genders (males, 60.7%; females, 50%). One-third (32.5%) disagreed with their gender IAT results, while the remaining 10% felt neutral.

**Table 2:** Self-reported result in Implicit Association Tests

Gender IAT	N	%
Strong automatic association of Male with Science and Female with Liberal Arts	4	10.3%
Moderate automatic association of Male with Science and Female with Liberal Arts	9	23.1%
Slight automatic association of Male with Science and Female with Liberal Arts	10	25.6%
Little to no automatic association between gender and academic domains	9	23.1%
Slight automatic association of Male with Liberal Arts and Female with Science	5	12.8%
Moderate automatic association of Male with Liberal Arts and Female with Science	1	2.6%
Strong automatic association of Male with Liberal Arts and Female with Science	1	2.6%
Race/Ethnicity IAT	N	%
Strong automatic preference for European American compared to African American	1	2.5%
Moderate automatic preference for European American compared to African American	9	22.5%
Slight automatic preference for European American compared to African American	8	20.0%
Little to no automatic preference between African American and European American	14	35.0%
Slight automatic preference for African American compared to European American	4	10.0%
Moderate automatic preference for African American compared to European American	2	5.0%
Strong automatic preference for African American compared to European American	2	5.0%

Race IAT results

Nearly half of respondents (45%) reported a race IAT result demonstrating some degree of automatic preference for white individuals, while 20% reported an automatic preference for black people. The remaining 35% reported no automatic preference (see Table 2). The distribution of responses for the race IAT did not differ between those who had previously taken an IAT and those who had not (p=0.50). Only 40% of faculty reported agreeing with their results on the race IAT, while 25% felt neutral, and just over a third (35%) disagreed with their results.

Perceived role of unconscious biases in faculty opportunities, evaluation, and promotion

**Figure 1:**
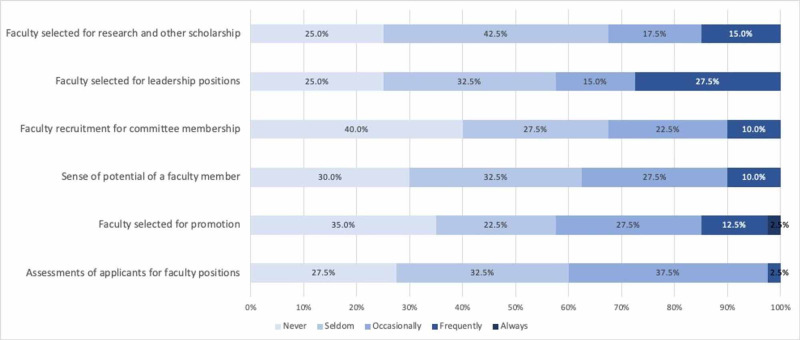
Perceived frequency of unconscious gender bias affecting faculty opportunities, evaluation, and promotion Data label = % of respondents in each category

**Figure 2:**
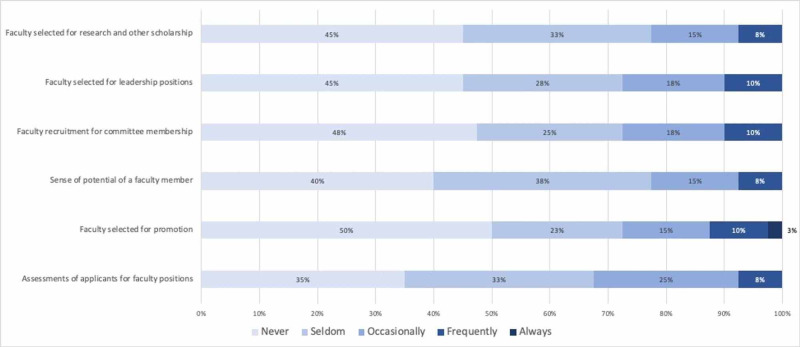
Perceived frequency of unconscious racial bias affecting faculty opportunities, evaluation, and promotion Data label = % of respondents in each category

**Figure 3:**
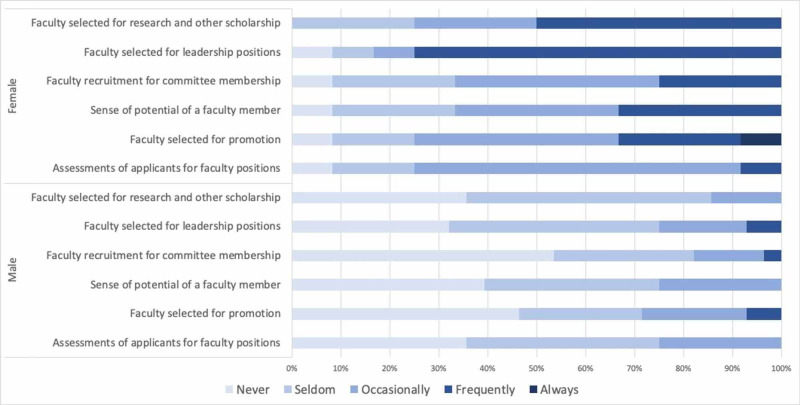
Perceived frequency of unconscious gender bias affecting faculty opportunities, evaluation, and promotion, by gender

We also elicited faculty perceptions regarding the frequency with which unconscious gender and racial bias affect faculty opportunities, evaluation, and promotion within our department (Figures 1 and 2). Most respondents (between 57% and 67% depending on the specific question) reported that unconscious gender bias never or seldom affects these areas (see Figure 1). Similarly, more than two-thirds of respondents felt that unconscious racial bias never or seldom affect these faculty-related factors (See Figure 2). However, when examined by the gender of the respondent, female faculty more frequently perceived that gender bias affects assessment of faculty applicants (p=0.004), faculty selected for promotion (p=0.003), perceptions of faculty members’ potential for success (p=0.003), as well as faculty recruited for committee positions (p=0.001), leadership positions (p<0.001) and collaborative research or other scholarship (p>0.001) (see Figure 3). The number of faculty respondents with URiM backgrounds as defined by the Association of American Medical Colleges (AAMC) was not sufficient to allow a similar analysis with relation to unconscious race bias. Interestingly, perceptions of racial bias affecting these decisions were significantly more common among female faculty compared to male faculty in each of these areas (applicant evaluation, p=0.003; promotion, p=0.002; perceived potential, p=0.001; recruitment for committees, p=0.001; leadership, p<0.001; research and other scholarship, p<0.001).

Comparison of IAT results to the general population

**Figure 4:**
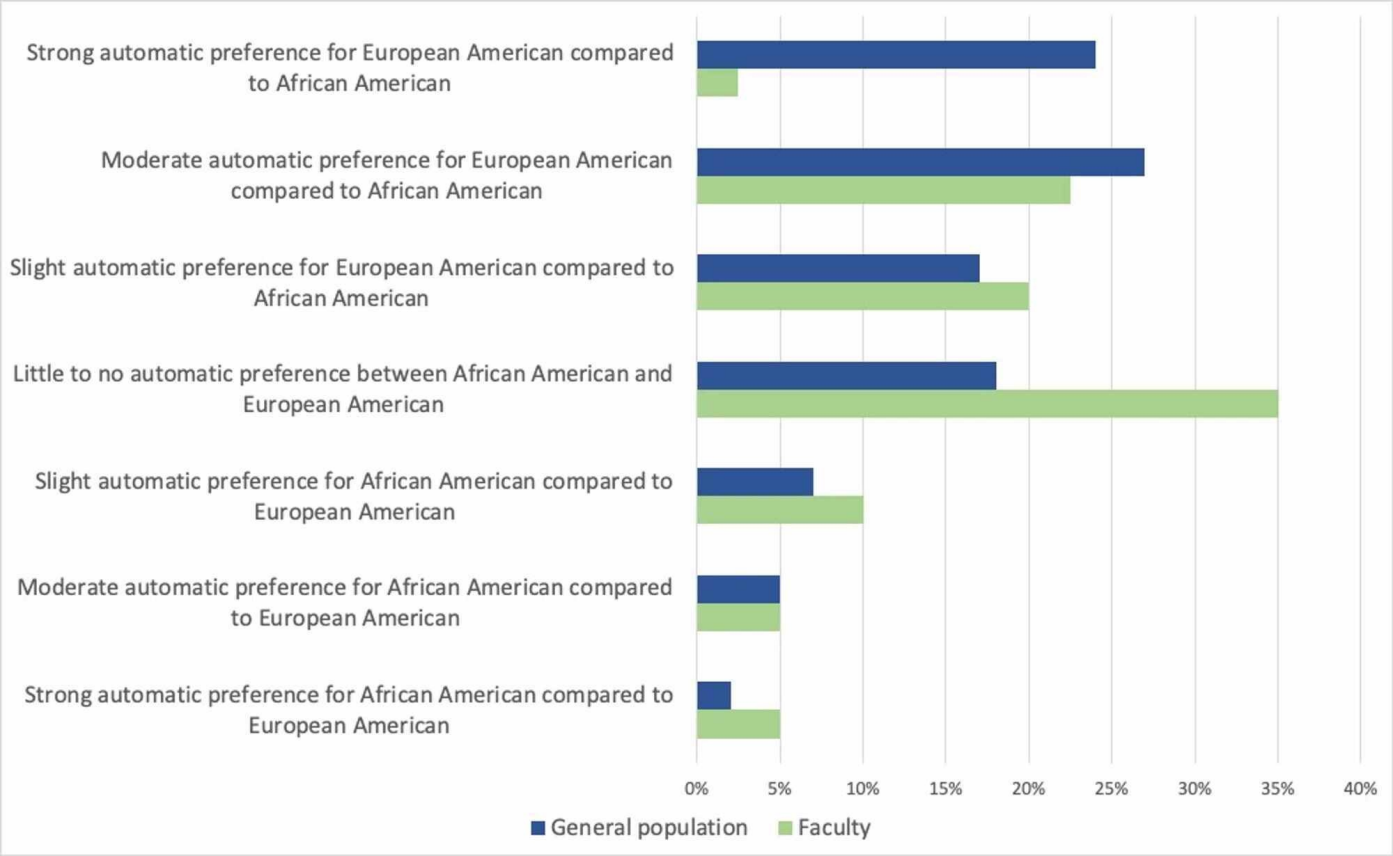
Comparison of Race IAT results between the Faculty and the General Population

On the gender IAT, the distribution of reported results among faculty differed somewhat from that of the general population (Figure 4). Overall, 70 percent of the general population respondents demonstrate some degree of automatic association between the male gender and the sciences, compared to roughly 60 percent of our faculty. Just over 17% of faculty respondents reported the opposite automatic association - that is, female with sciences - compared to 11% of the general population. Our faculty reported significantly less automatic association between the male gender and the sciences compared to the general population (p=0.02).

**Figure 5:**
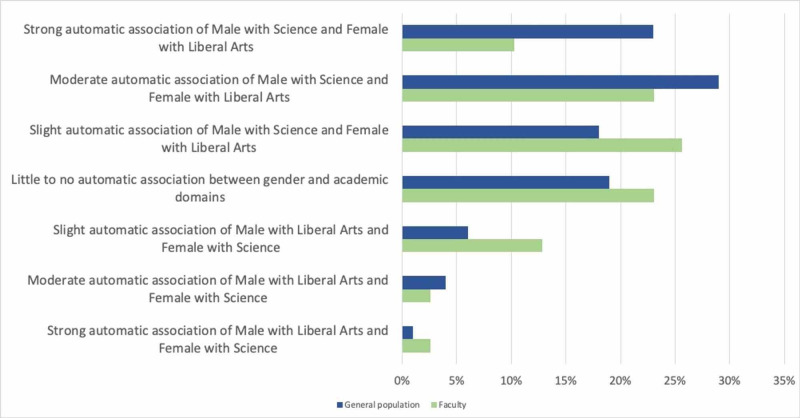
Comparison of Gender IAT results between the Faculty and the General Population

The distribution of our faculty’s self-reported results on the race IAT also differs from that of the overall population (Figure 5). Composite scores of the general population taking this IAT demonstrate that 68 percent have some degree of automatic preference for white faces, with just over half having a strong or moderate automatic preference, compared to 45 percent of the faculty respondents. Only 14 percent of the general population show an automatic preference for African Americans, while 20 percent of faculty respondents reported this result. When comparing the distribution of responses between faculty and the general population, faculty reported a lesser degree of automatic preference for European Americans than the general population (p<0.001).

## Discussion

Unconscious biases, also known as implicit biases, influence our thoughts, decisions, and behaviors without our conscious awareness, and may run contrary to our explicitly stated beliefs [1,3]. Implicit bias related to race and gender is not unique to any particular discipline or specialty in medicine, and prior studies have demonstrated that physicians have similar levels of implicit bias as people in the general population [20]. Pattern recognition is an integral aspect of providing clinical care in medicine, and particularly in the ED where clinicians often make decisions with limited information. However, unconscious biases can adversely impact health by contributing to inequities in clinical care and outcomes among patients from historically and contemporarily marginalized racial and ethnic groups [2,17,19]. Similarly, implicit biases contribute to disparities in employment opportunities for members of underrepresented racial and ethnic groups [21]. This may also be true in academic medicine, particularly for women and URiM faculty. This study sought to use IATs to prompt self-reflection and discussion of the role that gender and race play in shaping the clinical and academic work environment at our institution. In this effort, we also explored the extent to which unconscious racial and gender biases exist within our faculty group.

Inequality in the workplace adversely affects every member of the organization to a greater or lesser degree, including members of traditionally more powerful and advantaged groups. Higher levels of both implicit and explicit bias are correlated with higher rates of burnout [22], suggesting that biases foster a negative experience of the workplace. Unfairness in the workplace contributes substantially to members of the workforce leaving their positions, costing organizations and individuals both financial and emotional repercussions [23]. The toll on members of URiM groups is even more substantial, as these individuals experience both overt and subtle forms of discrimination in the form of microaggressions during medical training [12,13] and beyond [24,25].

Events outside the workplace may also disproportionately affect women and URiM faculty, the effects of which may be exacerbated if workplaces are not fully inclusive. In recent months, the economic and workplace disruption caused by the SARS-CoV-2 pandemic has led to significant changes in the burden of home-based responsibilities, such as for childcare and eldercare. The reported decrease in manuscript submission to major peer-reviewed journals by female researchers relative to male authors is anecdotal [26,27], but may reflect the disproportionate burden of non-professional responsibilities born by women during months when schools and other community supports were closed amidst the COVID-19 pandemic. Similarly, as COVID-19 has disproportionately affected communities of color, and as the deaths of black men and women while in police custody have sparked protests across the country, URiM faculty may be asked to contextualize these events in ways that are emotionally challenging. Amidst the subsequently renewed interest in racial equity and social justice efforts at many academic institutions, URiM faculty may again be asked to contribute both personal and professional time to help shape departmental and institutional responses. This manifestation of the minority tax may be compounded by working with colleagues who are less well versed in racial and social justice efforts, where implicit bias and microaggressions may emerge more frequently. The extent to which implicit bias contributes to burnout and discrimination in the workplace is not known, nor is the degree to which implicit bias affects decision-making related to academic promotion.

In an effort to promote self-reflection and to lay the groundwork for sensitive conversations in a professional setting, this study used IATs to frame questions and discussion related to workplace diversity and inclusion. Our findings demonstrate that our faculty group was largely willing to participate in IATs and discussions of sensitive topics including race and gender in the workplace (66% response rate) without any additional financial or otherwise concrete incentive. The distribution of our groups’ results on the two assigned IATs suggests that our faculty may have less unconscious preference for white individuals than the general population (p<0.001), and weaker implicit associations between male gender and the sciences (p=0.02). The fact that approximately a third of respondents reported disagreement with their race (35%) and gender (32.5%) IAT results underscores the reality that these tests identify unconscious associations that can be markedly discordant from our explicitly held beliefs. There is evidence, albeit limited, that simply taking an IAT may mitigate unconscious bias [28], perhaps by allowing people to intentionally counter unconscious thought patterns. This may have affected IAT results for the 20% of respondents who had previously taken an IAT before participating in this study. Other interventions to counter unconscious bias have shown positive results through exposure to positive exemplars of disadvantaged groups [29], as well as longer-term interventions that treat these thought patterns as habits, which can be broken through conscious effort, learning, and practice [30]. It is not known whether or the extent to which any of these interventions affect patient outcomes or patient perceptions of their clinical care.

Perceptions of whether unconscious bias influences decisions related to recruitment and promotion of faculty differed between male and female respondents. More than 70% of males reported that gender “never” or “seldom” influences any of the professional decisions explored in this study (see Table 3), and more than 80% of males reported that race never or seldom affects these domains. Females more frequently reported that gender bias plays a role in each of these areas (Table 3). Women also more frequently perceived that race affects decisions related to faculty recruitment and advancement. These findings suggest, perhaps unsurprisingly, that our female faculty members, regardless of race and ethnicity, are more sensitive to the ways in which multiple forms of discrimination manifest in the workplace. Further engagement is needed to understand the ways in which implicit bias and other forms of discrimination adversely affect departmental culture and academic advancement.

Limitations

This study has several important limitations. Firstly, this was conducted at a single site, thus our findings may not reflect the perceptions of bias at other institutions, particularly those with a more diverse workforce. Participation in this study was voluntary, and the two-thirds of faculty who completed the survey may differ from those who chose not to do so. However, respondent gender and race did mirror the overall demographic makeup of our department’s faculty. While we assume that faculty accurately reported their IAT results, all responses were self-reported anonymously and were not independently verified. The distribution of reported responses to the IATs in our faculty group differed from that of the general population. As these differences skewed away from the less socially acceptable biases (i.e., automatic preference for white faces and automatic associations between male gender and the sciences), our group’s reported results may reflect social desirability bias, whereby participants minimize or modify their responses to provide a response they perceive as more acceptable. Unconscious associations develop as a result of societal norms and messaging over the course of our lives, and thus reflect the cultural context in which we live, rather than individual beliefs. Respondents may nonetheless have been more reluctant to report IAT results that they perceived as suggestive of some level of harbored racism and/or sexism, especially when such results ran counter to their explicit beliefs and even when reporting anonymously.

## Conclusions

This study and the associated departmental efforts to improve faculty DE&I demonstrated the feasibility of using IAT as a tool for stimulating self-reflection and discussion among academic emergency physicians. Our group of academic physicians report having IAT results showing different levels of implicit bias compared to the general population. Female faculty may be both more aware of and more susceptible to the adverse effects of unconscious biases. Further study is needed to determine both the extent to which unconscious biases affect the academic workplace, as well as ways in which such unintentional forms of discrimination can be eliminated. Unconscious biases are not unique to EM. Intentional efforts to increase self-awareness of these 'blind spots' may help mitigate their impact and foster a more diverse and inclusive healthcare environment.
